# Pattern of dyslipidemia and associated factors in coronary artery disease patients in Khyber Pakhtunkhwa: A cross-sectional secondary data analysis

**DOI:** 10.12669/pjms.39.5.7382

**Published:** 2023

**Authors:** Arif Hussain, Muhammad Zakria, Iftikhar Ali, Shafiq Ahmad Tariq, Arshad Hussain, Sami Siraj

**Affiliations:** 1 Arif Hussain, Pharm D, MPhil, PhD scholar Institute of Pharmaceutical Sciences,Khyber Medical University, Peshawar, Pakistan; 2 Dr. Muhammad Zakria, Pharm D, PhD Institute of Pharmaceutical Sciences,Khyber Medical University, Peshawar, Pakistan; 3 Iftikhar Ali, Pharm D, MPH. Pharmacist, Paraplegic Centre, Hayatabad Peshawar, Pakistan; 4 Prof. Dr. Shafiq Ahmad Tariq, B Pharm, MPhil, PhD. Institute of Pharmaceutical Sciences,Khyber Medical University, Peshawar, Pakistan; 5 Dr. Arshad Hussain, FRCP (Edinburg), FRCP (Glasgow) Consultant Endocrinologist, Department of Medicine, Northwest General hospital & Research Centre, Peshawar, Pakistan; 6 Dr. Sami Siraj, B Pharm, MPhil, PhD. Associate Professor, Institute of Pharmaceutical Sciences,Khyber Medical University, Peshawar, Pakistan

**Keywords:** Dyslipidemia, Cardiovascular disease, Plasma lipids, Pattern, Adults Pakistan

## Abstract

**Objectives:**

To assess the prevalence, pattern, and associated factors of dyslipidemia in patients with coronary artery disease (CAD) in the Northwest region of Pakistan.

**Method:**

A cross-sectional secondary data analysis was performed on CAD patients visiting cardiology clinics in selected hospitals from July to December 2019. A total of 362 patients were included via consecutive sampling. Dyslipidemia was operationalized according to the “National Cholesterol Education Program (NCEP ATP III) guidelines”.

**Results:**

Mixed dyslipidemia was recorded in 92.26% of the patients, while isolated dyslipidemia was observed in 5.24%. A high prevalence of combined dyslipidemia with increased LDL-C, TG, and low HDL-C was noted. Contrarily, elevated LDL-C was the commonest single lipid disorder (84.25%). Hypercholesterolemia was the least common disorder. Increasing BMI was found to be independently associated with hypercholesterolemia (OR: 1.19). Similarly, age (OR: 0.97) and being a rural resident (OR: 2.61) were independent factors associated with hypertriglyceridemia. Furthermore, being an urban resident (OR: 2.25) and increasing BMI (OR: 1.77) were also significantly associated with high LDL-C.

**Conclusion:**

Mixed dyslipidemias were observed in the majority of the patients. Age, BMI, and residence were noted to be independently associated with abnormal lipids. Early screening and proper management should be encouraged to minimize this significant cardiovascular risk.

## INTRODUCTION

Cardiovascular diseases (CVD) comprise one of the main causes of mortality and disability-adjusted life years globally. As of 2019, nearly 17.90 million deaths have been attributed to CVD around the globe, accounting for one-third of all-cause mortality. The burden of CVD has almost doubled from 1990 to 2019.^1^ Although age-standardized CVD-related deaths have declined in developed countries, an increasing trend has been observed in developing nations.^2^,^3^

The most recognized predisposing factors for CVD include advanced age, male sex, smoking, hypertension, abnormal lipids, obesity, and a sedentary lifestyle,^4^-^6^ all of which are highly prevalent in South Asians with associated atherosclerotic cardiovascular disease,^3^,^7^ even in the absence of traditional risk factors.^8^ Lipid abnormalities have been reported to account for 2.6 million deaths annually, with a growing prevalence of 37% in males and 40% in females.^9^ A recent study from Iran described the prevalence of dyslipidemia among adults as 83% and 87% in the general population and CVD patients, respectively.^10^

In Pakistan, a 95.2% prevalence of dyslipidemia in the general population has been reported, and low HDL-C was observed in 87.4% of the population^11^, raising questions about such a disproportionately high prevalence in the country compared to other countries.^4^,^12^ With the ever-growing reports on the excess burden of CVD and the associated risk of dyslipidemia in this region, it is imperative to conduct studies to help establish specific management goals. The current study was conducted to assess the prevalence, patterns of dyslipidemia, and associated factors among patients with CAD in the Northwestern region of Pakistan.

## METHODS

This cross-sectional secondary data analysis was performed on patients newly diagnosed with CAD after coronary angiography, which is considered the gold standard for diagnosing patients visiting cardiology units at Hayatabad Medical Complex, Peshawar, District Headquarters (DHQ) Hospital Batkhela, and DHQ Parachinar from July to December 2019. A total of 362 people who had not yet received any therapeutic interventions were included via consecutive sampling. Patients with a major change in weight or diet intake or receiving lipid-lowering therapy, severe liver or renal disease, and thyroid dysregulation were excluded.

### Ethical Approval:

The Ethical Review Board of Khyber Medical University approved the study (Ref. # DIR/KMU-EB/AL000518; Date: 11-10-2018). BMI cutoffs were considered as defined by the “World Health Organization (WHO).^13^ TC, HDL-C, LDL-C, and TG levels were assayed through an automated biochemistry analyzer (Cobas c111, Roche, Germany). Non-HDL-C was determined by subtracting HDL-C from TC. Total ApoB was estimated using the following equations: “ApoB = 0.65 × TC−0.59 × HDL-C + 0.01 × TG” for TG < 270 mg/dl, while “ApoB = 25.6 + 0.58 × TC−0.38 × HDL-C−0.06 × TG” if TG > 270 mg/dl.^14^ Dyslipidemia was operationalized using the NCEP ATP III guidelines.^15^ Changes in two or more lipid parameters were considered mixed dyslipidemia.

The data were analyzed using SPSS V 23.0. Associations between the categorical variables were determined using x^2^ statistics or the Fisher exact test, where appropriate. The mean comparison was made using the independent t-test and one-way ANOVA. A logistic regression was performed to determine the factors independently associated with dyslipidemia. Statistical significance was established using a 95% Cl and an alpha of 0.05.

## RESULTS

The mean age of the patients was 59.14±7.98 years. Nearly 57% of the patients were male, while Afghan patients represented 3.04% of the total patients. About two-thirds of the patients were rural residents. BMI was calculated to be 27.25±2.54 kg/m^2^. Based on the “WHO” cutoffs, the majority 254(70.16%) were overweight. Background characteristics of the patients were stratified according to gender. Dyslipidemia was significantly (p=0.006) prevalent in rural male patients (Table-I).

**Table-I:** Background characteristics of the study participants.

Characteristics		Total	Female N=156	Male N=206	p-value
Age years (mean ± SD)		59.14±7.98	58.58 ±8.04	59.56±7.93	0.247
Age groups [n (%)]	44-55 years	153(42.30)	69(44.23)	84(40.78)	0.557
	56-67 years	162(44.80)	70(44.87)	92(44.66)	
	68-89 years	47(13.0)	17(10.90)	30(14.56)	
Nationality [n (%)]	Afghanistan	11(3.04)	2(1.28)	9(4.37)	0.124
	Pakistan	351(97.96)	154 (98.72)	197(95.63)	
Residence [n (%)]	Urban	129(35.64)	43 (27.56)	86(41.75)	0.006
	Rural	233(64.36)	113 (72.44)	120(58.25)
Weight (kg) (mean ± SD)		79.00±8.09	74.73±7.50	82.23±6.9	<0.001
Height (m) (mean ± SD)		1.70±0.06	1.65±0.04	1.74 ±0.04	<0.001
Body mass index (BMI) (mean ± SD)		27.25±2.54	27.36±2.76	27.16±2.37	0.664
BMI Cutoffs World Health Organization [n (%)]	Normal	65(17.96)	35(22.44)	30(14.60)	0.051
	Overweight	254(70.16)	99(63.46)	155(75.20)	
	Obese	43(11.88)	22(14.10)	21(10.20)	

The mean TC, HDL-C, TG, and LDL-C were observed to be 205±32.41 mg/dl, 34.93±7.32 mg/dl, and 203.61±83.20 mg/dl, respectively. Abnormal LDL-C was found in 84.25% of the patients, followed by HDL-C in 79.83%. Similarly, hypertriglyceridemia was observed in 72.09%, while hypercholesterolemia was observed in half the patients (Table-II).

**Table-II:** Age-and sex-specific prevalence of single and mixed dyslipidemia in the study population.

Lipids	Cutoffs N		Total population N (%) Age groups (Years)			Male N (%) Age groups (Years)			Female N (%) Age groups (Years)		
			44-55	56-67	68-89	44-55	56-67	68-89	44-55	56-67	68-89
TC	<200	181	73(47.71)	80(49.38)	28(59.57)	44(52.38)	53(57.61)	16(53.33)	29(42.03)	27(38.57)	12(70.59)
	≥200	181	80(52.29)	82(50.62)	19(40.43)	40(47.62)	39(42.39)	14(46.67)	40(57.97)	43(61.43)	5(29.41)*
HDL-C	>40	73	25(16.34)	36(22.22)	12(25.53)	9(10.71)	19(20.65)	8(26.67)	16(23.19)	17(24.29)	4(23.53)
	≤40	289	128(83.66)	126(77.78)	35(74.47)	75(89.29)	73(79.35)	22(73.33)	53(76.81)	53(75.71)	13(76.47)
TG	<150	101	37(24.18)	45(27.78)	19(40.43)	18(21.43)	30(32.61)	12(40.00)	19(27.54)	15(21.43)	7(41.18)
	≥150	261	116(75.82)	117(72.22)	28(59.57)	66(78.57)	62(67.39)	18(60.00)	50(72.46)	55(78.57)	10(58.82)
LDL-C	<100	57	24 (15.69)	21(12.96)	12(25.53)	11(13.10)	14(15.22)	6(20.00)	13(18.84)	7(10.00)	6(35.29)
	≥100	305	129 (84.31)	141(87.04)	35(74.47)	73(86.90)	78(84.78)	24(80.00)	56(81.16)	63(90.00)	11 (64.71)
TC/HDL-C Ratio	<5	76	30(19.61)	33(20.37)	13(27.66)	13(15.48)	18(19.57)	6(20.00)	17(24.64)	15(21.43)	7(41.18)
	≥5	286	123(80.39)	129(79.63)	34(72.34)	71(84.52)	74(80.43)	24(80.00)	52(75.36)	55(78.57)	10(58.82)
Non-HDL-C	<130	12	4(2.61)	5(3.09)	3(6.38)	3(3.57)	4(4.35)	2(6.67)	1(1.45)	1(1.43)	1(5.88)
	≥130	350	149(97.39)	157(96.91)	44(93.62)	81(96.43)	88(95.65)	28(93.33)	68(98.55)	69(98.57)	16(94.12)*
ApoB	<90	28	12(7.84)	12(7.41)	11(23.4)	6(7.14)	10(10.87)	6(20.00)	6(8.70)	2(2.86)	5(29.41)
	≥90	334	141(92.16)	150(92.59)	36(76.6)	78(92.86)	82(89.13)	24(80.00)	63(91.3)	68(97.14)	12(70.59)*
Mixed dyslipidemia	No	28	12(7.84)	10(6.17)	6(12.77	5(5.95)	8(8.70)	3(10.00)	7(10.14)	2(2.86)	3(17.65)
	Yes	334	141(92.16)	152(93.83)	41(87.23)	79(94.05)	84(91.30)	27(90.00)	62(89.86)	68(97.14)	14(82.35)

* p<0.05

**Fig.1:**
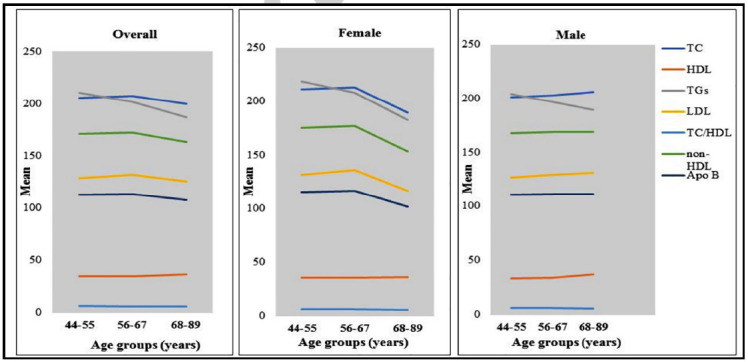
Mean levels of serum lipid components with age. The group means of male and female patients, as well as the variations between age groups, were compared using the student’s t-test and an ANOVA with pairwise analysis, respectively.

Among the lipid parameters, only the TC level was observed to be higher in females compared to males (p = 0.039). With increasing age, however, a slight decrease in the TG and improvement in the HDL-C were noted, while these changes were more prominent in males than in females, whereas in females, lipid parameters except for HDL-C and the TC/HDL-C ratio were either unchanged or decreased with age, and there was a significant difference between age groups and TC, non-HDL-C, and ApoB in females. (Fig.1 and Table-II).

**Fig.2:**
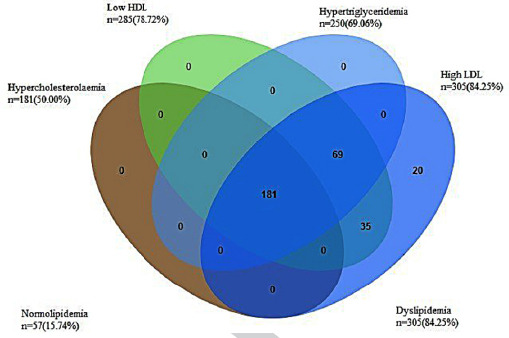
Venn diagram depicting the overlapping components of abnormal lipids (elevated TC, LDL-C, and TG, and low HDL-C)

Abnormal LDL-C was the most prevalent single lipid disorder 305(84.25%), while hypercholesterolemia was the least common 181(50.00%) in the study patients. The overall prevalence for the abnormal lipid parameters is shown in Fig.2.

A significant difference in TC levels among males and females was observed. Similarly, TC differed across the BMI groups; obese patients had higher TC than normal and overweight patients. Moreover, the TG level considerably differed between urban and rural residents (p<0.001). Furthermore, LDL, TC/HDL-C ratio, non-HDL, and ApoB in obese patients compared to normal and overweight patients had higher mean levels (Table-III).

**Table-III:** Mean lipids parameters, their ratio, and frequency of mixed dyslipidemia based on various background characteristics.

Characteristics		TC	HDL-C		TG		LDL-C	TC/HDL-C	Non-HDL-C	ApoB	Mixed dyslipidemia	
		Mean± SD	Mean± SD		Mean± SD		Mean± SD	Mean± SD	Mean± SD	Mean± SD	No	Yes
Age Groups	44-55 years	205.56± 31.65	34.45± 7.51	210.8± 82.6		128.95± 31.96	6.24± 1.65	171.1± 32.57		112.67± 19.99	12(7.84)	141(92.16)
	56-67 years	207.38± 33.13	34.87± 6.92	201.67± 84.49		132.18± 29.68	6.16± 1.47	172.51± 32.84		113.59± 20.27	10(6.17)	152(93.83)
	68-89 years	199.96± 32.38	36.77± 7.93	186.94± 79.54		125.8± 34.57	5.66± 1.4	163.19± 32.45		107.9± 20.2	6(12.77	41(87.23)
Gender	Female	209.69± 32.66	35.72± 7.29	210.22± 85.83		131.92± 31.97	6.12± 1.63	173.97± 33.18		114.48± 20.41	12(42.86)	144(43.11)
	Male	202.58*± 31.96	34.34± 7.31	198.62± 81		128.51± 30.79	6.13± 1.49	168.24± 32.24		110.93± 19.9	16(57.14)	190(56.89)
Nationality	Afghanistan	192.18± 26.84	32.64± 5.85	217.27± 72.19		116.09± 32.92	6.06± 1.28	159.55± 26.93		105.87± 17.04	0	11(3.29)
	Pakistan	206.07± 32.51	35.01± 7.36	203.19± 83.58		130.42± 31.2	6.13± 1.56	171.06± 32.86		112.67± 20.24	28(100.00)	323(96.7)
Residence	Urban	204.74± 32.97	34.6± 7.35	181.76**± 84.51		133.8± 30.65	6.18± 1.64	170.15± 33.65		112.02± 20.73	11(39.29)	118(35.33)
	Rural	206.15± 32.16	35.13± 7.32	215.72± 80.12		127.87± 31.53	6.10± 1.50	171.02± 32.27		112.71± 19.89	17(60.71)	216(64.67)
BMI Cutoffs	Normal	198.74*± 34.95	34.71± 9.22	209.74± 83.08		122.08*± 33.96	6.09± 1.87	164.03*± 36.66		108.18*± 22.39	8(28.57)	57(17.07)
	Overweight	204.93± 30.58	35.23± 6.78	198.69± 81.56		129.96± 30.12	6.01*± 1.4	169.69± 30.4		111.96± 18.9	17(60.71)	237(70.96)
	Obese	220.35± 35.16	33.56± 7.22	223.47± 91.13		142.1± 30.9	6.84± 1.68	186.79± 35.34		121.9± 21.42	3(10.71)	40(11.98)

* p<0.05,

** p<0.001.

Logistic regression analysis showed that increasing BMI was found to be independently associated with hypercholesterolemia [OR: 1.19(95%Cl) 1.09-1.30, p<0.001]. Similarly, a decreasing age [OR: 0.97 (95%Cl) 0.94 -1.00, p =0.020] and being a rural resident (OR: 2.61(95%Cl) 1.60-4.25, p <0.001) was independently associated with hypertriglyceridemia. Likewise, being an urban resident (OR: 2.25 (95% Cl) 1.13-5.88, p =0.024) and increase in BMI (OR: 1.77 (95%Cl) 1.03-1.35, p =0.018) were also significantly associated with high LDL-C. Increasing BMI was found to be associated with a high TC/HDL-C ratio (p=0.025) and high non-HDL-C (p=0.04). Similarly, decreasing age (p=0.01), and increasing BMI (p<0.019) were found to be significantly associated with high ApoB.

## DISCUSSION

The proportion of patients with mixed dyslipidemia was 92.26% in this study, with high LDL-C, low HDL-C, and elevated TG. This pattern of significantly low HDL-C and high TG levels has extensively been described as a risk factor for CVD in SAs.^7^,^11^,^16^ Our study showed that 20.16% of the patients had normal HDL-C levels; hence, such patients can be classified as low-risk for CAD. Similar findings were observed by Zaid M and Hasnain S in a local study.^17^

The determinants of CAD, especially dyslipidemia, show considerable variations. In SAs expatriates in Saudi Arabia, CAD was found to be associated with predominant low levels of HDL-C and elevated TG.^18^ A remarkably high prevalence of low HDL-C was also reported in the Asia-Pacific region^19^, supporting the higher prevalence of dyslipidemia in SAs. Smoking, low literacy, obesity, and low HDL-C in a rural Chinese population predicted increased CVD risk.^20^ The frequency of high TG (28-72.2%), low HDL-C (27-72.2%), high LDL-C(23.3-44.5%), and high TC (19-38.7%) was reported in the Indian population.^4^

In a comparable Indian population with CAD, Sharma SB^21^ has reported a high prevalence of raised TG (72.2%) and low HDL-C (72.2%), with TC (29%), which is consistent to with observations. High TG and low HDL-C were observed in 69.06% of patients in our study. Comparing elevated TG and small-dense low-density lipoprotein with low HDL-C to elevated LDL-C and TC with low HDL-C, Western studies highlighted the significance of these differences in the development of CAD.^4^,^5^ This emphasizes the significance of low HDL-C and high TG in the Pakistani population relative to the Western population as CVD risk factors, which others have also addressed.^11^,^17^

There was no significant difference in lipid parameters among males and females except for high TC in females, which is in concordance with the published literature on Pakistani and Indian populations. Although the majority of CAD risk factors are the same for both sexes, there are a few cases where they differ. Moreover, studies have found that women have higher amounts of atherogenic lipids.^22^,^23^ However, such different results might have been attributed to variations in the study population’s characteristics. The gender-wise calculation revealed that such age-specific changes in serum lipid parameters were more noticeable in males compared to females. A significant difference in lipid parameters observed between males and females across different age groups, particularly the major drop in lipid levels among women in the oldest age group, is indeed an intriguing finding. Some of the possible explanations could be related to hormonal changes associated with menopause, lifestyle factors, differences in disease prevalence, and genetic and epigenetic factors.

The increasing trend of dyslipidemia in urban areas is evident from the existing literature.^17^ A high proportion of people with abnormal lipids living in urban areas compared to rural areas except for hypercholesterolemia, has been reported earlier.^11^ The mean levels of TC and HDL-C were higher in rural residents compared to urban residents, similar to those of the NDSP.^11^ Similarly, significantly higher TGs were observed in rural residents compared to urban residents. In contrast, the prevalence of hypertriglyceridemia was almost the same in urban and rural populations of all age groups reported in the NDSP.

The proportion of mixed dyslipidemia was higher in rural as compared to urban areas. This seems to be in contrast to other local studies.^11^,^17^ Still, this rural inclination in our study could be attributed to further heterogeneity in the rural population, lack of access to healthcare, lack of awareness, and consequently poor dietary consciousness and physical activity, as reported by Harrington et al.,^24^with a rural prevalence of CHD at 14.2% against the urban areas, 11.2%. This higher prevalence of risk factors, especially dyslipidemia, in rural CHD residents has also been reported by Hosseini et al.,^25^ in the Iranian population. Although a drop has been observed in CHD-related deaths in urban areas, the same cannot always be extrapolated to rural areas, as mentioned in the US rural health asseement.^24^

Similarly, obese individual had higher mean levels of TC, LDL-C, TG, TC/HDL-C ratio, and low HDL-C. Similar findings were also noted by Zahid N et al.^26^ and strong correlation between dyslipidemia and adiposity was observed by Souza et al.^27^ Obesity is linked to an increased risk of CAD, diabetes, dyslipidemia, and hypertension.^28^ In NHS data, lean (BMI <21) women had a 4-fold lower CAD mortality rate than obese women.^29^ BMI, and abdominal obesity are correlated with dyslipidemia.

### Limitations:

Between the BMI groups, a sizable mean difference in TC and LDL was noted. Since this is secondary data analysis, only considering a cross-sectional view of the patients at baseline due to dropping out of the participants, all the limitations of such studies could be expected in this study. Additionally, other variables, including medicine, waist circumference, diet, lifestyle habits, and chronic comorbidity, may have confounded the results.

## CONCLUSION

Mixed dyslipidemia was observed in the majority of the patients. High LDL-C was the commonest single lipid disorder, while hypercholesterolemia was the least common disorder. Combined dyslipidemia was low HDL-C, high LDL-C, and high TG. Factors found to be independently associated with abnormal lipids were age, BMI, and residence. Given the high burden of dyslipidemia, it is imperative to encourage early screening and proper management to minimize this significant cardiovascular risk.

### Authors’ Contributions:

**AH, SS, SAT & MZ:** Conception, design, data collection, and manuscript writing

**MZ, IA:** Data analysis, interpretation/results and initial drafting

**AH, MZ & IA:** Manuscript drafting and writing.

**AH, MZ, SS,, AH, SAT & IA:** Language editing, critical revision.

All authors have read and approved the final version of the paper. The principal investigator is responsible and accountable for the accuracy or integrity of the work.
